# Changes in occupational class differences in leisure-time physical activity: a follow-up study

**DOI:** 10.1186/1479-5868-8-14

**Published:** 2011-03-01

**Authors:** Tina Seiluri, Jouni Lahti, Ossi Rahkonen, Eero Lahelma, Tea Lallukka

**Affiliations:** 1 Hjelt Institute, Department of Public Health, University of Helsinki, Helsinki, Finland

## Abstract

**Background:**

Physical activity is known to have health benefits across population groups. However, less is known about changes over time in socioeconomic differences in leisure-time physical activity and the reasons for the changes. We hypothesised that class differences in leisure-time physical activity would widen over time due to declining physical activity among the lower occupational classes. We examined whether occupational class differences in leisure-time physical activity change over time in a cohort of Finnish middle-aged women and men. We also examined whether a set of selected covariates could account for the observed changes.

**Methods:**

The data were derived from the Helsinki Health Study cohort mail surveys; the respondents were 40-60-year-old employees of the City of Helsinki at baseline in 2000-2002 (n = 8960, response rate 67%). Follow-up questionnaires were sent to the baseline respondents in 2007 (n = 7332, response rate 83%). The outcome measure was leisure-time physical activity, including commuting, converted to metabolic equivalent tasks (MET). Socioeconomic position was measured by occupational class (professionals, semi-professionals, routine non-manual employees and manual workers). The covariates included baseline age, marital status, limiting long-lasting illness, common mental disorders, job strain, physical and mental health functioning, smoking, body mass index, and employment status at follow-up. Firstly the analyses focused on changes over time in age adjusted prevalence of leisure-time physical activity. Secondly, logistic regression analysis was used to adjust for covariates of changes in occupational class differences in leisure-time physical activity.

**Results:**

At baseline there were no occupational class differences in leisure-time physical activity. Over the follow-up leisure-time physical activity increased among those in the higher classes and decreased among manual workers, suggesting the emergence of occupational class differences at follow-up. Women in routine non-manual and manual classes and men in the manual class tended to be more often physically inactive in their leisure-time (<14 MET hours/week) and to be less often active (>30 MET hours/week) than those in the top two classes. Adjustment for the covariates did not substantially affect the observed occupational class differences in leisure-time physical activity at follow-up.

**Conclusions:**

Occupational class differences in leisure-time physical activity emerged over the follow-up period among both women and men. Leisure-time physical activity needs to be promoted among ageing employees, especially among manual workers.

## Background

Health behaviours, such as leisure-time physical activity tend to be socioeconomically patterned. Such patterning is complex as socioeconomic position covers a range of social, economic and material circumstances from childhood to adulthood [1]. The main subdomains of socioeconomic position include education, occupational class and income [2]. While the subdomains are correlated with each other, they nevertheless are not interchangeable. Occupational class is a key subdomain of socioeconomic position and particularly suitable when an occupational cohorts are studied.

Cross-sectional studies suggest that people in higher education [3,4] and occupational class [5] are more often physically active in their leisure time than counterparts in lower positions. There has been a tendency in Finland over the last few decades for those on lower income levels to be less physically active in their leisure time than those with higher incomes [6]. However, female manual workers were found to engage in higher levels of active commuting than those in higher occupational classes [6].

In the last few decades, increases in leisure-time physical activity have been reported in Finland [6], Canada [7] and the USA [8]. The prevalence of adult Finns engaging in leisure-time physical activity at least twice a week increased from 37% to 55% among women and from 38% to 62% among men between 1978-2002 [6]. In Canada, the prevalence of adults who were physically active in leisure-time increased from 20% to 40% between 1981-2000 [7]. In the USA, the prevalence of those who were physically inactive in their leisure-time declined during 1988-2008 from 31% to 25% [8]. In Australia, the prevalence of those who were physically inactive in leisure-time has been remained almost stable [9].

According to a study on trends in Finland, education, occupational class and household income differences in leisure-time and commuting physical activity generally remained relatively small between 1978-2002 [6]. Follow-up studies examining changes over time in physical activity by socioeconomic position have been conducted in the Netherlands [10,11] and Denmark [12]. Those with lower education were more likely to reduce their leisure-time physical activity during follow-up [10] whereas those with higher education were more likely to remain active than their lower education counterparts [11,12].

It is known that low leisure-time physical activity in general is associated with higher weight [3,11,13], smoking [3,11], poorer physical health functioning [14], mental health [15], being married [11] and being unmarried [16]. Retirement has been associated with increasing physical activity [17]. It has been suggested that those in lower occupational classes or those with lower education have more often poor health [18] and therefore are less likely to be physically active than those in higher classes [19]. Only few previous studies have considered covariates for socioeconomic differences in physical activity or their changes over time. In a previous study occupational class differences in leisure-time physical inactivity were attenuated after taking job strain, physical workload, body mass index (BMI) and smoking into account [20].

We hypothesised that class differences in leisure-time physical activity would widen over time due to declining physical activity among the lower occupational classes. Our first aim was to examine occupational class differences in leisure-time physical activity, and subsequent changes, over a follow-up of 5 to 7 years. Our second aim was to examine the effect of covariates on changes in the occupational class differences in leisure-time physical activity.

## Methods

### Data

The data were derived from the Helsinki Health Study cohort mail questionnaire surveys administered to employees of the City of Helsinki, Finland. At baseline in 2000-2002, the data covered 8 960 employees aged 40-60-years (response rate of 67%, 80% of the respondents were women) [21]. The follow-up survey was conducted in 2007 among the respondents to the baseline survey. At follow-up, there were 7 332 respondents, which gives a response rate of 83%. The baseline data is broadly representative of the target population and the non-response is unlikely to bias the relationships between socioeconomic position and the health-related variables [22]. Our control analyses showed that non-respondents to the follow-up were only slightly more physically inactive (<14 MET hours/week) during leisure-time at baseline (among women 24% 95% CI 22.9-25.1 vs. 29% 95% CI 26.1-31.3). However, occupational class differences in leisure-time physical activity among the respondents and the non-respondents, were broadly similar.

The analyses for this study were carried out among those with data on occupational class (missing data for 118), leisure-time physical activity (missing data for 135) and all the covariates (missing data for 123). After exclusions for missing data, the final data consisted of 5 652 women and 1 279 men.

The Helsinki Health Study protocol has been approved by ethics committees of the Department of Public Health, University of Helsinki, and the City of Helsinki health authorities, Finland.

### Leisure-time physical activity

The respondents were asked to estimate the average weekly hours of physical activity during their leisure time (including commuting) within the previous year [14]. There were four levels of intensity: walking, brisk walking, jogging and running, or their equivalent activities. The response alternatives for each level were: not during the past twelve months, in total under half an hour per week, between half and one hour per week, between two and three hours per week, and four hours or more per week.

The amount of leisure-time physical activity was assessed by approximate metabolic equivalent tasks (MET) taking into account the intensity, duration and frequency [23] and calculated by multiplying the weekly time used by the estimated MET value of each activity level [14,24].

Moderate-intensity leisure-time physical activity for about 30 minutes at least five times a week is recommended by the national guidelines in Finland as well as other countries [25-27]. Approximately 14 MET hours per week correspond to the energy expenditure (1000 kcal, e.g. brisk walking for 2.5 hours/week equals 15 MET hours) needed for reducing health risks associated with physical inactivity [13,28-30]. An optimal 30 MET hours fulfill the recommendations [27,31] and requirements for healthy weight maintenance [32]. We use the term physically inactive for under 14 MET hours per week, and physically active for over 30 MET hours.

### Socioeconomic position

Occupational class was used as an indicator of socioeconomic position. Information on occupational class was derived from the City of Helsinki personnel registers for those who gave written permission for data linkage (77%) [21]. For the rest, occupational class was obtained from the questionnaires. The respondents were classified into four hierarchical occupational classes: professionals including managers, semi-professionals, routine non-manual employees and manual workers. Among women, 27% were professionals, 20% semi-professionals, 39% routine non-manual employees and 14% manual workers. The corresponding figures for men were 46%, 20%, 10% and 24%.

### Covariates

All the covariates were self-reported and taken from the baseline survey, except employment status which was taken from the follow-up survey. Thirteen per cent of women were single, 10% cohabiting, 58% married, 16% divorced and 3% were widowed. The corresponding figures for men were 11%, 15%, 64%, 10% and 1%. Twenty-two per cent of women and 25 per cent of men were smokers at baseline. Body mass index (BMI) was calculated as the weight in kilograms divided by the height in metres squared (kg/m^2^) (the means were 25.3 and 26.3 kg/m^2 ^for women and men, respectively). Physical and mental health functioning were measured on the physical component summary (PCS) and the mental component summary (MCS) of the Short Form 36 (SF-36) questionnaire [33]. The average PCS score was 48.9 among women and 50.8 among men, and the respective MCS scores were 51.7 and 51.7. Lower scores indicate poorer health functioning. Both were dichotomised by the lowest quartile. At follow-up most of the respondents were still in employment (75% of women and 71% of men), but some had retired due to disability (4%) or old age (18% and 23%, respectively).

### Statistical analyses

All the analyses were carried out separately for women and men. First, the age-adjusted prevalence and 95% confidence intervals of the physically inactive and active participants at baseline and follow-up were calculated by occupational class. Logistic regression analysis was then used to examine the emerging occupational differences in leisure-time physical activity adjusting for covariates. Model 1 is adjusted for age, and model 2 for age and baseline leisure-time physical activity. Further models additionally adjusted for baseline marital status, smoking, BMI, mental and physical health functioning, and employment status one covariate at a time. The SPSS (version 15.0) was used for the analyses.

## Results

At baseline 24% of women were physically inactive and there were no differences between the occupational classes (Table 1). The prevalence of physical inactivity at follow-up was 22%, but the higher classes were less likely to be physically inactive than their lower class counterparts. At follow-up, fewer professionals (19%) and semi-professionals (18%) than routine non-manual employees (24%) and manual workers (27%) were physically inactive. Professionals increased their leisure-time physical activity to the recommended level, whereas the level of inactivity in the other classes remained almost stable. Thus there were occupational class differences at follow-up suggesting a gradient.

**Table 1:** Age-adjusted prevalence (%) and 95% confidence intervals (95% CI) for physically inactive and active women and men at baseline and at follow-up.

	**Physically inactive, <14 MET**^**1 **^**hours/week**					**Physically active, >30 MET**^**1 **^**hours/week**				
	**Baseline**		**Follow-up**			**Baseline**		**Follow-up**		
	**%**	**95% CI**	**%**	**95% CI**	**Change %-unit**	**%**	**95% CI**	**%**	**95% CI**	**Change %-unit**

Women										
Professionals (n = 1524)	24	[21.4-25.7]	19	[17.2-21.1]	-5	35	[32.7-37.4]	42	[39.3-44.2]	+7
Semi-professionals (n = 1138)	21	[18.7-23.4]	18	[16.0-20.5]	-3	39	[36.2-42.0]	44	[41.5-47.3]	+5
Routine non-manual employees (n = 2227)	25	[23.3-26.8]	24	[22.1-25.7]	-1	36	[34.1-38.0]	36	[34.3-38.2]	0
Manual workers (n = 763)	26	[23.2-29.4]	27	[24.3-30.6]	+1	37	[33.1-39.9]	33	[30.0-36.6]	-4
										
Total (n = 5652)	24	[22.9-25.1]	22	[20.9-23.1]	-2	37	[35.2-37.8]	39	[37.7-40.3]	+2
										
Men	%		%			%		%		
Professionals (n = 584)	20	[17.1-23.6]	20	[16.6-23.1]	0	46	[41.6-49.7]	49	[45.0-53.1]	+3
Semi-professionals (n = 257)	29	[23.4-34.6]	22	[16.8-26.8]	-7	42	[36.0-48.2]	45	[39.3-51.4]	+3
Routine non-manual employees (n = 128)	27	[19.7-35.1]	21	[13.7-27.7]	-6	42	[33.0-50.0]	43	[34.8-51.9]	+1
Manual workers (n = 310)	28	[23.3-33.3]	36	[30.3-40.9]	+8	40	[34.9-45.7]	31	[26.1-36.4]	-9
										
Total (n = 1279)	25	[22.3-27.1]	24	[21.9-26.5]	-1	43	[40.5-45.9]	43	[40.7-46.1]	+0

^1^MET = an activity metabolic equivalent task

Thirty-seven percent of women were physically active in their leisure-time at baseline and there were no differences between the occupational classes (Table 1). At follow-up 39% were physically active. However, more among the professionals (42%) and semi-professionals (44%) than among routine non-manual employees (36%) and manual workers (33%) were physically active. The prevalence of physically active increased among the professionals and semi-professionals and remained stable or decreased in the lower classes, thus leading to socioeconomic differences over the follow-up.

Twenty-five per cent of men were physically inactive at baseline and as with women there were no differences between the occupational classes (Table 1). At follow-up the prevalence of inactivity was 24%, but the higher classes tended to be less often physically inactive than their manual worker counterparts in leisure-time. The prevalence of physical inactivity among male manual workers increased by eight percentage points (from 28% to 36%) leading to higher prevalence than in the other occupational classes. Among the semi-professionals (from 29% to 22%) and routine non-manual employees (from 27% to 21%) it decreased over the follow-up. Thus there were occupational class differences in leisure-time physical activity at the follow-up.

Among men 43% were physically active during their leisure-time and there were no differences between the occupational classes at baseline (Table 1). Among male manual workers the prevalence of physically active decreased during the follow-up by 9 percentage units (from 40% to 31%) whereas the higher classes somewhat increased their leisure-time physical activity leading to occupational class differences at follow-up.

Next logistic regression analysis was used to examine the effects of the covariates on the occupational class differences in leisure-time physical activity at follow-up. Firstly, we examined physical inactivity. Among women, after adjusting for age, manual workers (OR 1.60 95% CI 1.31-1.97) and routine non-manual employees (OR 1.34 95% CI 1.14-1.57) were more likely to be physically inactive at follow-up than professionals (Table 2). We further examined the emergence of occupational class differences in leisure-time physical activity by adjusting for baseline physical activity and it did not change the associations. Of the baseline covariates only BMI (OR for manual workers 1.47 95% CI 1.18-1.83 and for routine non-manual employees 1.27 95% CI 1.07-1.50) slightly attenuated the association.

**Table 2:** Odds ratios (OR) and their 95% confidence intervals (95% CI) among women (n = 5652) and men (n = 1279). Physically inactive (<14 MET^1 ^hours/week) at follow-up.

	**Model 1**^**2**^		**Model 2**^**3**^		Model 2 + marital status		Model 2 + smoking		**Model 2 + BMI**^**4**^		**Model 2 + PCS**^**5**^		**Model 2 + MCS**^**6**^		Model 2 + employment status	
Women	OR	CI 95%	OR	95% CI	OR	95% CI	OR	95% CI	OR	95% CI	OR	95% CI	OR	95% CI	OR	95% CI
Professionals (n = 1524)	1.00		1.00		1.00		1.00		1.00		1.00		1.00		1.00	
Semi-professionals (n = 1138)	0.95	[0.78-1.16]	1.00	[0.81-1.23]	1.00	[0.81-1.23]	0.99	[0.81-1.22]	0.96	[0.78-1.19]	0.98	[0.80-1.21]	1.01	[0.82-1.24]	0.98	[0.80-1.21]
Routine non-manual employees (n = 2227)	1.34	[1.14-1.57]	1.39	[1.17-1.64]	1.37	[1.16-1.63]	1.34	[1.13-1.59]	1.27	[1.07-1.50]	1.34	[1.13-1.58]	1.41	[1.19-1.67]	1.35	[1.14-1.59]
Manual workers (n = 763)	1.60	[1.31-1.97]	1.65	[1.33-2.05]	1.62	[1.31-2.02]	1.56	[1.25-1.93]	1.47	[1.18-1.83]	1.59	[1.28-1.97]	1.69	[1.36-2.10]	1.58	[1.27-1.96]
																
Men	OR	CI 95%	OR	CI 95%	OR	CI 95%	OR	CI 95%	OR	CI 95%	OR	CI 95%	OR	CI 95%	OR	CI 95%
Professionals (n = 584)	1.00		1.00		1.00		1.00		1.00		1.00		1.00		1.00	
Semi-professionals (n = 257)	1.12	[0.78-1.60]	1.04	[0.72-1.51]	1.02	[0.70-1.48]	1.03	[0.71-1.50]	0.97	[0.67-1.42]	1.01	[0.69-1.46]	1.04	[0.72-1.52]	1.04	[0.71-1.51]
Routine non-manual employees (n = 128)	1.03	[0.64-1.67]	0.97	[0.59-1.61]	0.88	[0.53-1.46]	0.91	[0.55-1.51]	0.89	[0.53-1.47]	0.93	[0.56-1.54]	0.98	[0.59-1.61]	0.98	[0.59-1.62]
Manual workers (n = 310)	2.21	[1.62-3.02]	2.19	[1.58-3.04]	2.04	[1.47-2.85]	2.12	[1.52-2.94]	2.04	[1.47-2.84]	2.11	[1.52-2.94]	2.21	[1.59-3.07]	2.22	[1.59-3.08]

^1^MET = an activity metabolic equivalent task

^2^Model 1 = adjusted for age

^3^Model 2 = adjusted for age and baseline physical activity

^4^BMI = body mass index

^5^PCS = SF-36 physical component summary

^6^MCS = SF-36 mental component summary

Secondly, we examined the physically active. Among women, after adjusting for age, manual workers (OR 0.69 95% CI 0.57-0.83) and routine non-manual employees (OR 0.79 95% CI 0.69-0.91) were less likely to be physically active at follow-up than professionals (Table 3). Adjusting for baseline physical activity did not change the associations, whereas BMI had a slight attenuating effect on the studied association.

**Table 3:** Odds ratios (OR) and their 95% confidence intervals (95% CI) among women (n = 5652) and men (n = 1279). Physically active (>30 MET^1 ^hours/week) at follow-up.

	**Model 1**^**2**^		**Model 2**^**3**^		Model 2 + marital status		Model 2 + smoking		**Model 2 + BMI**^**4**^		**Model 2 + PCS**^**5**^		**Model 2 + MCS**^**6**^		Model 2 + employment status	
**Women**	**OR**	**CI 95%**	**OR**	**95% CI**	**OR**	**95% CI**	**OR**	**95% CI**	**OR**	**95% CI**	**OR**	**95% CI**	**OR**	**95% CI**	**OR**	**95% CI**
Professionals (n = 1524)	1.00		1.00		1.00		1.00		1.00		1.00		1.00		1.00	
Semi-professionals (n= 1138)	1.12	[0.95-1.30]	1.06	[0.90-1.25]	1.06	[0.90-1.26]	1.07	[0.90-1.26]	1.11	[0.94-1.32]	1.08	[0.91-1.27]	1.06	[0.90-1.25]	1.07	[0.90-1.26]
Routine non-manual employees (n = 2227)	0.79	[0.69-0.91]	0.75	[0.65-0.86]	0.76	[0.65-0.87]	0.76	[0.66-0.88]	0.82	[0.71-0.95]	0.77	[0.67-0.89]	0.74	[0.64-0.86]	0.76	[0.66-0.88]
Manual workers (n = 763)	0.69	[0.57-0.83]	0.65	[0.54-0.79]	0.66	[0.54-0.80]	0.67	[0.55-0.82]	0.74	[0.61-0.90]	0.67	[0.55-0.82]	0.64	[0.53-0.78]	0.67	[0.55-0.82]
																
Men	OR	CI 95%	OR	CI 95%	OR	CI 95%	OR	CI 95%	OR	CI 95%	OR	CI 95%	OR	CI 95%	OR	CI 95%
Professionals (n = 584)	1.00		1.00		1.00		1.00		1.00		1.00		1.00		1.00	
Semi-professionals (n = 257)	0.86	[0.64-1.15]	0.90	[0.65-1.25]	0.89	[0.65-1.24]	0.92	[0.66-1.27]	0.93	[0.67-1.29]	0.91	[0.66-1.26]	0.90	[0.65-1.25]	0.92	[0.66-1.27]
Routine non-manual employees (n = 128)	0.79	[0.54-1.17]	0.81	[0.53-1.24]	0.83	[0.54-1.28]	0.90	[0.58-1.39]	0.85	[0.55-1.31]	0.81	[0.53-1.25]	0.81	[0.53-1.24]	0.76	[0.49-1.16]
Manual workers (n = 310)	0.47	[0.35-0.63]	0.42	[0.30-0.59]	0.43	[0.31-0.60]	0.44	[0.32-0.62]	0.44	[0.32-0.62]	0.43	[0.31-0.59]	0.42	[0.30-0.59]	0.40	[0.29-0.55]

^1^MET = an activity metabolic equivalent task

^2^Model 1 = adjusted for age

^3^Model 2 = adjusted for age and baseline physical activity

^4^BMI = body mass index

^5^PCS = SF-36 physical component summary

^6^MCS = SF-36 mental component summary

Among men, after adjusting for age, manual workers (OR 2.21 95% CI 1.62-3.02) were more likely to be physically inactive at follow-up than those in the upper classes (Table 2). Adjusting for baseline physical activity did not affect the association. Of the covariates only marital status (OR 2.04 95% CI 1.47-2.85) and BMI (OR 2.04 95% CI 1.47-2.84) slightly attenuated the differences between the manual workers and the upper classes.

Among men, after adjusting for age manual workers (OR 0.47 95% CI 0.35-0.63) were less likely to be physically active at follow-up than those in the upper classes (Table 3). Adjusting for baseline physical activity (OR 0.42 95% CI 0.30-0.59) had virtually no effect on the associations.

## Discussion

### Main findings

The focus in this study was on occupational class differences in leisure-time physical activity among middle-aged women and men over a follow-up of 5 to 7 years. A further aim was to find out which covariates would affect such differences. We hypothesised that class differences in leisure-time physical activity would widen over time. The results showed that there were no considerable class differences in leisure time physical activity at baseline, but hierarchical occupational class differences in leisure-time physical activity emerged over the follow-up. In women the levels of leisure-time physical activity increased among the professionals and semi-professionals, and decreased among the manual workers. Among male manual workers there was a substantial decrease in leisure-time physical activity. None of the examined social or health-related baseline covariates or employment status at follow-up affected substantially the occupational class differences in leisure-time physical activity at follow-up.

### Interpretation

In the whole sample, level of leisure-time physical activity remained stable over the 5 to 7 years follow-up. Some previous trend studies have reported an increase in leisure-time physical activity [6,7,34]. As found in many other studies [7,35] here, too, men were more physically active than women. Occupational differences in leisure-time physical activity emerged at follow-up, with those in the upper classes being more physically active than their lower class counterparts. The decrease in leisure-time physical activity among manual workers we observed is consistent with the findings of Danish [11] and a Dutch study [10]. Another Dutch study [12] found that those in higher positions remained physically active over time.

Our findings revealed that occupational class differences in leisure-time physical activity, which did not exist at baseline, emerged during the follow-up. The data do not show when such differences developed. In principle they might have existed even before the baseline, disappeared and then appeared again at follow-up. However this is unlikely, particularly given the results of a Finnish trend study showing that socioeconomic differences in physical activity have largely remained relatively small in recent decades [6]. Most of our participants work in permanent jobs for the same employer and share many similar circumstances such as occupational health care. However, over the follow-up almost a third exit workforce e.g. due to retirement. Among the ageing employees occupational class differences in health widen [36], potentially contributing to the emergence of class differences in physical activity as well.

In our ageing cohort a fifth retired during the follow-up. Retirement is a major life transition that may affect health behaviours including leisure-time physical activity [17]. Also other life events could contribute to physical activity and the emergence of socioeconomic differences. Adjusting for employment status, however, did not affect the observed differences.

Those in the higher occupational classes and with higher education have better knowledge and they are more ready to adopt healthier behaviours and reduce risk behaviours than those in lower classes [19]. This might explain why the higher classes increased their leisure-time physical activity. It is an unfortunate development that manual workers engage less in leisure-time physical activity as they age. People who are less physically active benefit most from following the recommended level of physical activity [28].

None of the baseline covariates we examined had a substantial effect on the occupational class differences found at follow-up. We also took into consideration health behaviours, sociodemographic factors as well as physical and mental health. We controlled not only for the factors included in the reported analyses but also for the effects of limiting long-lasting illness, common mental disorders and job strain on the identified socioeconomic differences in leisure-time physical activity (data not shown). However none of these additional covariates had a substantial effect on the emergent occupational class differences. We also checked the effects of covariates measured at follow-up but the results were similar to those using covariates measured at baseline.

Occupational class differences in health and functioning tend to widen towards late middle-age, with manual workers' health being the worst [36]. Health problems that also restrict physical activity during leisure-time may occur even more among the lower occupational classes. In this study the association of occupational class and leisure-time physical activity, however, did not attenuate after adjusting for the physical component summary (PCS) of the SF-36 health inventory.

Therefore we conducted control analyses and adjusted for the physical functioning subscale of the SF-36 which more precisely measures health problems that restrict physical activities such as running and brisk walking. The physical functioning subscale is by definition a purer measure of physical functioning than the physical component summary which is a composite measure [33]. The differences in physical functioning may be partly masked when using the physical component summary.

Further control analyses showed that the follow-up physical functioning score attenuated the association more than the other examined covariates (data not shown), however, the baseline adjustment had no effects. This suggests that there were differences in physical functioning between the occupational classes at follow-up that affect leisure-time physical activity. In other words leisure-time physical activity decreased among the lower occupational classes partly due to poorer health as suggested by previous research [18]. Our single follow-up design and use of logistic regression analysis, however, do not allow causal interpretations or pathways between the exposure, outcome and control variables. The question why occupational class differences in leisure-time physical activity emerged remains open to further scrutiny.

One also might ask whether similar developments could be observed in other health behaviours. With regard to the consumption of healthy food, for example, the socioeconomic gap has remained stable, and food habits increasingly tend to follow national guidelines [37]. Further studies are needed to examine the importance of these and other factors for the socioeconomic differences in leisure-time physical activity and the underlying mechanisms.

### Methodological considerations

Given that our study was based on an occupational cohort we used occupational class for the analyses, which reflects material resources and working conditions, in particular. We used education as a parallel socioeconomic indicator in our control analyses. Education is an indicator reflecting knowledge and affecting unhealthy behaviours. However, the results of the control analyses were similar to those reported above (data not shown).

The baseline survey was conducted in spring and the follow-up in autumn, but because physical activity was measured over the previous year, there should be no seasonal effects on the results. In this study leisure-time physical activity was self-reported, which is common practice in epidemiological studies [10,34,35]. Physical activity may be overestimated in surveys, but among adults the reporting has not been biased by education or gender [38]. We acknowledge that the measurement of leisure-time physical activity is a complicated task, and a recent review concluded that there is no golden standard for measuring physical activity in questionnaires and no single measure has proven superior [39].

We examined leisure-time physical activity while some other studies have also examined occupational physical activity. Working life has changed over the decades, and is generally less physically demanding [40]. This is the case in Finland, but there has nevertheless been an increase in physical activity during leisure-time overall [6]. Manual workers do more physically strenuous work than other classes and this may lead to less leisure-time physical activity, especially if problems of health and functioning have emerged along ageing. Regarding male occupations approximately half of male manual workers are public transport drivers implying sedentary work.

The response rate was acceptable both at baseline (67%) and follow-up (83%), but non-participation was still a concern and may have biased the findings. Our analysis of the baseline non-response indicated that the associations in question were unlikely to be severely affected [22]. According to our further control analyses bias due to attrition is unlikely to substantially affect the studied associations.

All the respondents were from the Helsinki metropolitan area and employed at baseline by the City of Helsinki. The results therefore cannot be generalised to the whole population of Finland, and not even to the employed population at large. Nevertheless, the cohort is large and diverse, and the study was planned to enable studies of the socioeconomic differences in lifestyles and health. A further strength was the use of identical questions at baseline and follow-up in assessing occupational class differences in leisure-time physical activity.

## Conclusions

Occupational class differences in leisure-time physical activity emerged during the follow-up of 5 to 7 years: there was an increase in activity among the upper classes and a decrease among the lower classes. In the interests of health promotion and disease prevention it is important for ageing people of all occupational classes, but especially for manual workers to maintain and increase physical activity. Efforts should also be made to reduce the socioeconomic differences. In the future mechanisms behind the socioeconomic differences in leisure-time physical activity should be further examined.

## List of abbreviations

BMI: body mass index; MCS: the mental component summary; MET: an activity metabolic equivalent task; PCS: the physical component summary; SF-36: the Short Form 36;

## Competing interests

The authors declare that they have no competing interests.

## Authors' contributions

TS carried out the statistical analysis, interpreted the results and drafted the manuscript. TS, JL, TL, OR and EL contributed to designing the study, interpreting the results and drafting the manuscript. All the authors critically reviewed the manuscript and approved the final version.
